# *Pseudoalteromonas* Strains as Biofilm Control Agents in *Ostrea edulis* Aquaculture: Reducing Biofilm Biovolume While Preserving Microbial Diversity

**DOI:** 10.3390/microorganisms13020363

**Published:** 2025-02-07

**Authors:** Garance Leroy, Leila Parizadeh, Héléna Cuny, Clément Offret, Martin Protat, Alexis Bazire, Sophie Rodrigues, Patrick Le Chevalier, Benjamin Brillet, Ricardo Gonzalez-Araya, Camille Jégou, Yannick Fleury

**Affiliations:** 1 Université de Brest, CNRS EMR 6076, IUEM, LBCM, F-29000 Quimper, France; 2 Comité Régional de la Conchyliculture de Bretagne-Nord (CRC BN), F-29678 Morlaix, France; 3 Universite Bretagne Sud, CNRS EMR 6076, IUEM, LBCM, F-56100 Lorient, France

**Keywords:** *Pseudoalteromonas*, biofilms, microbiota, *Ostrea edulis*, aquaculture

## Abstract

Biofilms in aquaculture tanks pose significant challenges, hindering cleaning processes and contributing to antibiotic resistance. This study investigated the effects of four *Pseudoalteromonas* strains on flat oyster (*Ostrea edulis*) rearing, with a specific focus on biofilm control and microbial communities. After confirming the safety of these strains for *O. edulis*, we monitored biofilm development and bacterial communities during a 4-month sexual maturation period. Biofilm biovolume was quantified using confocal laser scanning microscopy (CLSM), and bacterial community composition was analyzed via 16S rRNA gene metabarcoding of both biofilm and seawater samples. Our results revealed differences in bacterial community structure between biofilms and seawater. Furthermore, the presence of specific *Pseudoalteromonas* strains significantly impacted the composition of bacterial communities within the tanks. β-diversity analyses demonstrated that each strain exerted a unique influence on the bacterial community structure. Some *Pseudoalteromonas* strains effectively reduced biofilm biovolume without negatively impacting bacterial richness or diversity. These observations suggest that certain *Pseudoalteromonas* strains can effectively control biofilm formation while maintaining a diverse and potentially beneficial microbial community in *O. edulis* rearing tanks. The use of these strains as additives in aquaculture systems could offer several advantages, including reduced cleaning time and costs and a potential decrease in biocide usage.

## 1. Introduction

One of the main challenges in the coming decades will consist of feeding 9 billion people by 2050 in the context of global climate change, which is recognized as a fundamental threat to the security of the food supply [1]. According to the FAO, aquaculture will play a strategic role in producing high-quality food, as well as ensuring economically and socially sustainable development [2]. In 2022, the global fishery and aquaculture production of aquatic animals reached an unprecedented volume of 185 million tons [3]. In France, shellfish farming is the most common form of aquaculture, with an oyster production of 80,943 tons in 2021 [4]. However, in recent years, aquaculture has been negatively affected by mortality events, resulting from different factors such as global warming [5] and the use of intensive breeding techniques [6]. Bacterial diseases cause massive mortalities among vertebrates [7] and invertebrates [8], responsible for considerable economic losses in aquaculture. To address this issue, the common strategy employed is the use of antibiotics as an attempt to limit the economic risks associated with bacterial infections [9]. The use of antibiotics is nonetheless associated with the emergence of resistant bacteria [10]. Moreover, the biofilms formed within aquaculture tanks exacerbate the phenomenon of antibiotic resistance as they persist and lead to horizontal transfers of antimicrobial resistance genes [11]. Bacterial biofilms are defined as microorganisms encased in an extracellular matrix (extracellular polymeric substance or EPS) composed of polysaccharides, proteins, nucleic acids (DNA, RNA), and other biomolecules [12]. Biofilms can harbor pathogenic strains and increase the risk of bacterial infections within aquaculture rearing [13]. Antibiofilm strategies are currently under development to limit the risks associated with the biofilm formation in the tanks. This can be achieved by modifying abiotic surfaces through antimicrobial peptide coating [14], modulating communication pathways with quorum sensing inhibitors [15,16], using dietary supplements such as phytogenic additives [17], or using probiotics [18].

Most of the probiotics currently used and tested in aquaculture are lactic acid bacteria *Bacillus* sp., *Bifidobacterium, Pseudomonas* sp., *Streptococcus* sp., *Saccharomyces cerevisiae*, or *Pseudoalteromonas* sp. [19,20]. These probiotics are mainly used to control disease, boost immune response, and provide nutritional and enzymatic contributions but can also improve water quality [21]. In this context, marine bacteria belonging to the genus *Pseudoalteromonas* are well known for their biotechnological potential, notably for their ability to produce antimicrobial [22] and antibiofilm compounds [23]. Previous studies led to the isolation of two bacterial strains from oyster hemolymph, both belonging to the genus *Pseudoalteromonas*, namely *Pseudoalteromonas ostreae* h*Oe*-125 [24] and *Pseudoalteromonas rhizosphaerae* h*Cg*-42 [25]. Both strains produce cationic cyclolipopeptides with in vitro antibacterial activity [26]. Another *Pseudoalteromonas* strain named *Pseudoalteromonas* sp. 3J6 was chosen for its capability to inhibit the in vitro biofilm development of various bacteria [27,28]. Treatments with *Pseudoalteromonas* strains h*Oe*-125, h*Cg*-42, and 3J6 have been shown to reduce the maximum thickness of biofilms in tanks used for juvenile sea bass rearing [29]. In this study, we investigated the potential of *Pseudoalteromonas* strains h*Cg*-42, h*Oe*-125, and 3J6 to limit biofilm development in oyster rearing. Biofilm bacterial communities were also studied to determine whether changes in biofilm biovolume were accompanied by variations in communities’ structure, richness, and diversity.

## 2. Materials and Methods

### 2.1. Bacterial Strains and Growth Conditions

*Pseudoalteromonas* strains h*Cg*-42, h*Oe*-125, and 3J6 were selected to evaluate their antibiofilm activities in vivo. The *Pseudoalteromonas* h*Cg*-42 and h*Oe*-125 strains were isolated in France from the hemolymph of healthy wild bivalves, *Crassostrea gigas* and *Ostrea edulis*, respectively [24,25]. The h*Cg*-42 and h*Oe*-125 strains are known for their production of antimicrobial peptides named alterins [30]. The *Pseudoalteromonas* 3J6 strain was isolated from a glass slide immersed in seawater (SW) at a depth of 1 m for 6 h in the Morbihan Gulf in France [27,31]. The 3J6 strain has previously been characterized for its in vitro antibiofilm activity, attributed to the production of an antibiofilm protein called alterocin [23]. *Pseudoalteromonas rhizosphaerae* RA15^T^ is not known to produce alterins or alterocin and was therefore used as a control strain [32]. The selected strains were cultivated in Marine Broth (MB) medium (Difco™ 2216, Thermo Fisher Scientific, Waltham, MA, USA) at 18 °C under agitation at 100 rpm for 48 h.

### 2.2. Preparation of Pseudoalteromonas Suspensions

*Pseudoalteromonas* strains were cultivated from one colony in 200 mL Erlenmeyer flasks containing 20 mL MB under stirring at 100 rpm for 48 h to yield pre-cultures. These were used to inoculate 1 L of MB medium poured into 2 L Erlenmeyer flasks, which were also incubated for 48 h under stirring. The bacterial cultures were centrifuged at 7000× *g* for 15 min (4 °C), and the culture supernatant was discarded to dispose of the culture medium. Next, the cellular pellet was resuspended in a sterile 3% SeaSalts (Sigma-Aldrich, Saint Louis, MO, USA) solution and then centrifuged at 7000× *g* for 15 min (4 °C). This washing step was performed twice. Finally, the bacterial cells were suspended in a 3% sterile SeaSalts solution. The bacterial concentration of the *Pseudoalteromonas* suspensions was determined by preparing serial dilutions ranging from 10^−1^ to 10^−8^ for each tested strain. When necessary, suspensions were diluted and the volume of bacterial inoculum was adjusted to reach a final concentration of 10^6^ UFC/mL in the tanks.

### 2.3. Flat Oyster Pre-Experimental Conditioning and Diet

Three-year-old flat oysters (*Ostrea edulis* (L.), *n* = 650, flesh dry weight = 0.5 g, length = 65 to 70 mm) were sampled from the roadstead of Brest (Brittany, France) and transferred into an experimental aquaculture platform (CRCBN, Lampaul-Plouarzel). Oysters were acclimated to the experimental seawater temperature (19 °C) by gradually increasing the temperature for 15 days. The oysters were fed a mixture of four phytoplankton species, namely *Cylindrotheca gracilis*, *Rhodomonas salina*, *Conticriba weissflogii*, and *Skeletonema marinoï* with a daily ration adjusted to 10^9^ cells per broodstock per day. Starting from the second month of conditioning, 10% of the microalga T-Iso (*Tisochrysis lutea* with a cell volume of approximately 40 µm^3^) was added to the base diet to ensure the availability of food that could be ingested by larvae within the oyster’s pallial cavity.

### 2.4. Sexual Maturation Experimental Design

**Figure 1:**
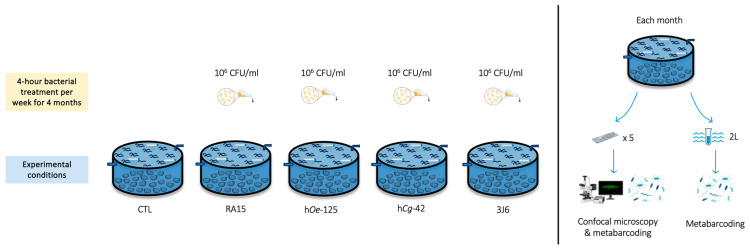
Experimental design of the flat oysters’ maturation at CRC Bretagne Nord.

Flat oysters (*n* = 100 per experimental condition) were distributed homogeneously in flow-through 480 L high-density polyethylene breeding tanks containing 100 L of seawater maintained at 19 °C with 34 ± 0.79 mg/L salinity. The seawater was pumped 600 m from the hatchery at the mouth of the Aber Ildut, starting two hours before high tide and up to a maximum of one hour after high tide. It then passed through a filtration system consisting of a sand filter, followed by a series of bag filters with pore sizes of 25, 10, 5, and 1 micron and finished with UV filters. All tanks were aerated, and the seawater was replenished by 20% every hour. Oysters bathing with *Pseudoalteromonas* strains (10^6^ CFU/mL) was performed for 4 h (interruption of seawater renewal) once a week from December 2022 to March 2023. Five experimental conditions were undertaken using the *Pseudoalteromonas* strains RA15^T^, h*Oe*-125, h*Cg*-42, and 3J6, along with a control (CTL) condition where no suspensions were added. Oysters’ mortality in each tank was monitored every day during the experiment (Figure 1).

To investigate the effects of *Pseudoalteromonas* strains on biofilm biovolume, microscope glass slides (76 × 26 mm, MGF) were immersed vertically in each tank for each condition (30 glass slides/experimental condition).

### 2.5. Biofilm and Seawater Sampling

Biofilm and seawater samples were collected throughout the 4-month experiments. Every month, a total of five glass slides and 2 L of SW were sampled in each tank for analysis. One side of the glass slide was retrieved using a Cell Scraper (Biologix Europe, Hallbergmoos, Germany), and the other side was used for CLSM analysis. Seawater samples were filtered through a 0.22 µm polycarbonate filter (Thermo Fisher Scientific, Whatman, MA, USA). Filters and scraped biofilms were stored at −80 °C prior to DNA extraction.

### 2.6. Confocal Laser Scanning Microscopy (CLSM)

#### 2.6.1. Glass Slides Treatment and Observation

Glass slides were rinsed three times in a sterile 3% SeaSalts solution and then dried in a dark at room temperature for 15 min. Subsequently, glass slides were stained with 50 μL of 5 μM SYTO9 green (Invitrogen, Thermo Fischer Scientific, Waltham, MA, USA) and incubated for 5 min. Glass slides were then rinsed once again before being treated with ProLong Diamond (Thermo Fischer Scientific, USA), allowing for the preservation of the slides before observation under the microscope. Biofilm observations were performed with a CLSM microscope (Zeiss, LSM710, Jena, Germay) using a 63× oil immersion objective. SYTO9 green was excited using a 488 nm filter, and fluorescence emission was detected between 500 and 550 nm. For the visualization of the three-dimensional (3D) structure, images were acquired every micrometer across the entire depth of the biofilm. At least five random images were taken from each of the five replicate glass slides, yielding a total of 25 image stacks per sampling day. Three-dimensional images were obtained and analyzed using Zeiss Zen Blue 2 lite software (Zeiss, Germany). Biofilm biovolume was determined with COMSTAT software (v.1 2000) [33].

#### 2.6.2. Statistical Analyses on Biofilm Biovolumes

Statistical analyses were performed using RStudio (v2023.12.1.402; [34]). A non-parametric Kruskal–Wallis test [35] was conducted, followed by Dunn’s multiple comparison test [36] to assess statistical differences.

### 2.7. 16S rRNA Gene Metabarcoding Analyses

#### 2.7.1. gDNA Extraction and Sequencing

Bacterial genomic DNA from biofilm samples and filters was extracted using Qiagen PowerBiofilm and PowerWater kits (Qiagen, Hilden, Germany), respectively, in accordance with the supplier’s instructions. Samples were sent for sequencing and analysis at Genome Quebec Innovation Centre (Montreal, QC, Canada). The amplicon libraries for each sample were generated using the forward primer 341F 5′-CCTACGGGNGGCWGCAG-3′ and reverse primer 805R 5′-GACTACHVGGGTATCTAATCC-3′ [37]. These primers target the V3–V4 region of the 16S rRNA gene amplicons. Sequencing was then performed using Illumina MiSeq PE300 (10 million read pairs per pooled library), (Illumina, San Diego, CA, USA).

#### 2.7.2. Bioinformatic Pipelines for 16S rRNA Gene Metabarcoding

The files obtained after sequencing were then analyzed using the Standardized and Automated Metabarcoding Analysis (SAMBA) workflow (v4.0.0; [38]), developed by Ifremer’s Bioinformatics Service. Primers were removed from the obtained sequences using the Cutadapt tool (v4.2; [39]) with an error rate of 1%. The expected amplicon size of 464 base pairs was specified in the pipeline’s configuration file. Sequencing data were processed with QIIME 2 (v2022.11; [40]), FIGARO (v1.1.2; [41]), and DADA2 (v1.26.0; [42]). FIGARO was used to adjust DADA2 parameters. The obtained amplicons (amplicon sequence variants or ASVs) were then grouped using the dbOTU3 tool (v1.5.3; [43]). Taxonomic assignment was performed by querying the SILVA database v138.1 (16S V3-V4 341F-805R). Phyloseq objects for statistical analyses were generated using the R phyloseq package (v1.46.0; [44]).

#### 2.7.3. Statistical Analyses

α-diversity analyses were conducted in RStudio (v2023.12.1.402) using the plot_richness function from the phyloseq package (v1.46.0; [44]). The Chao1, Shannon, and Inverse Simpson indices were calculated to assess the richness and diversity of samples under different treatments (exposures to bacterial strains within various tanks). An analysis of variance (ANOVA; [45]) was performed on these results using the anova_test function from the rstatix package [46]. Beforehand, the normality and the homogeneity of variances were assessed using the Shapiro–Wilk [47] and Levene [48] statistical tests from the package rstatix (v0.7.2). ASVs that accounted for 99.6% of total abundance in the samples were used for relative abundance analysis. Data were normalized using the cumulative sum scaling method (CSS; [49]) to allow for comparison between samples in the context of ß-diversity analyses. Graphical representations (principal coordinate analysis and non-metric multidimensional scaling) were generated using Bray–Curtis distance to observe differences between bacterial communities. The influence of the two sampling groups (seawater and biofilm) and various conditions was assessed through a permutation-based analysis of variance [50] (PERMANOVA) using the adonis2 function from the vegan package (v2.6-4; [51]). Linear discriminant analysis (LDA) was performed using the MicrobiotaProcess package (v1.15.0; [52]) to evaluate the differential abundance of taxa.

## 3. Results

### 3.1. Sequencing Data Information

After demultiplexing, 6,838,523 sequence reads were used for analyses with a mean of 53,848 sequences per sample (*n* = 120) observed. A total of 13,383 ASVs were inferred via the SAMBA pipeline and 7068 remained after clustering (52.8%). Biofilm samples (6523 ASVs) and seawater samples (1552 ASVs) shared 1007 identical ASVs. Rarefaction curves (Supplementary Data, Figure S1) indicate that the sequencing depth can be regarded as sufficient to fully explore the diversity of ASVs.

### 3.2. Bacterial Communities Associated with Flat Oyster Rearing

#### 3.2.1. Divergent Community Structure Between Seawater and Biofilm Samples

During the experimentation, bacterial communities from biofilms showed significantly higher richness than those from seawater (ANOVA test, *p*-value < 0.05; Supplementary Data, Table S1). An average of 316 ± 90 ASVs were identified in biofilm samples compared to 202 ± 54 ASVs in seawater samples from the control condition when pooling samples collected over the four-month period (Supplementary Data, Figure S2). ß-diversity analyses (Figure S3) also showed that the shaping of microbial communities significantly differed according to the sample origin (Biofilm vs. seawater, PERMANOVA test, R^2^ = 0.17, F = 4.65, *p*-value = 0.001).

#### 3.2.2. Temporal Dynamics of Environmental Bacterial Communities

**Figure 2:**
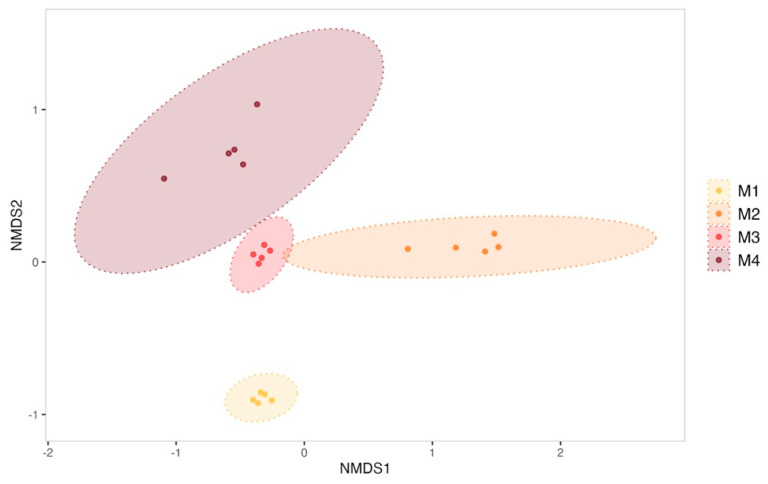
Non-metric multidimensional scaling (NMDS) of biofilm bacterial communities from the control condition sampled each month (M1 to M4). Ellipses were drawn at a 99% confidence level for a multivariate t-distribution. Stress value: 0.04631038.

The evolution of biofilms formed within the tanks was monitored over the 4-month sexual maturation of the flat oyster *Ostrea edulis*. Bacterial communities from biofilm were significantly enriched with new ASVs throughout the maturation (ANOVA test on observed richness, *p*-value < 0.05; Supplementary Data, Figure S4). For example, the bacterial community from the biofilm formed in the experimental control tank consisted of 263 ± 33 ASVs in the first month of the experiment, which increased significantly to 372 ± 122 ASVs in the fourth month (post hoc Tukey test on observed richness, *p*-value = 1.42 × 10^−4^). A significant increase in diversity was also observed in the control tank (ANOVA test on the Shannon and Simpson inverse indexes, *p*-value < 0.05; Supplementary Data, Figure S4). The Shannon index indeed started at 4.50 ± 0.13 in the first month and showed a significant decrease in the second month (3.86 ± 0.40, post hoc Tukey test, *p*-value = 0.0041). However, diversity increased significantly in the third and fourth months, reaching 5.14 ± 0.23 (post hoc Tukey test first month vs. fourth month, *p*-value = 0.00323) by the end of the experiment. On the other hand, ß-diversity analyses have revealed a significant shift in the structure of microbial communities (Figure 2) between all sampling months of biofilm samples from the control condition (PERMANOVA test, R^2^ = 0.70, F = 12.67, *p*-value = 0.001).

#### 3.2.3. Differential Taxonomic Composition of Biofilm and Seawater Samples

**Figure 3:**
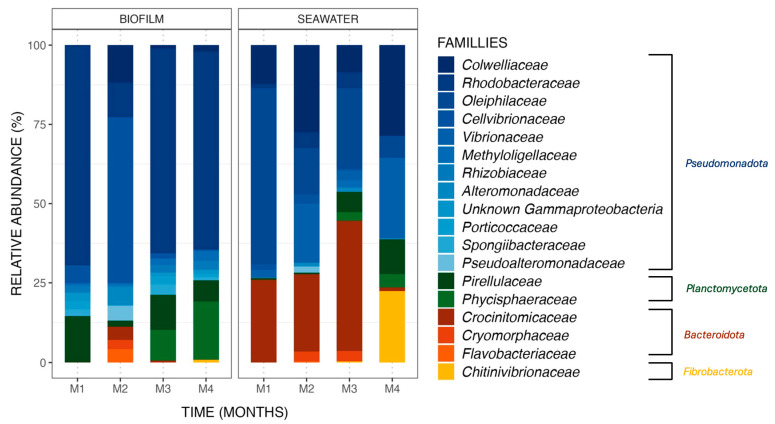
Relative abundance (%) of the major ASVs found in biofilm and seawater samples in the control condition at each sampling month (M1 to M4). ASVs are represented at the family taxonomic rank. Together, the ASVs shown here comprise 99.6% of all observed ASVs.

Bacterial communities differed between biofilm and seawater samples, with differences in (i) the nature of the ASV observed and (ii) their relative abundances. ASV (relative abundances > 0.4% of total sequences) found in biofilm and seawater of the control tank (Figure 3) belonged to four phyla, namely *Pseudomonadota*, *Planctomycoteta*, *Bacteroidota*, and *Fibrobacterota.* ASVs affiliated with the phyla *Pseudomonadota* were the most abundant in the bacterial communities of all samples from biofilm (81.3%) and seawater (63.2%). It is also noteworthy to observe that the phyla *Bacteroidota* and *Fibrobacterota* were found in seawater at higher relative abundances (24.8% and 5.7%, respectively) than in biofilms (3.0% and 0.2%).

Differences between biofilm and seawater samples regarding relative abundances were hence observed at the phylum level but were also noted at the family level. For example, the *Rhodobacteraceae* family dominated the biofilm bacterial communities, with an average relative abundance of 51.7% in the monthly samples from the control condition, a pattern not observed in the seawater samples with an average abundance value of 2.9%.

#### 3.2.4. Variations in Taxonomic Abundances Observed Throughout the 4-Month Experiment

Changes in taxonomic relative abundances were also observed within biofilm and seawater microbial communities for each sampling month. A gradual increase in the relative abundance of the *Phycisphaeraceae* family was observed in the biofilm samples, starting from 0.0%, then rising to 0.1%, 9.5%, and reaching 18.2% over the course of the 4-month experiment. Additionally, the *Chitinovibrionaceae* family (22.4%) was detected in seawater samples exclusively in the fourth month (Figure 3). Differential abundance analysis was performed to obtain further details on biofilm bacterial communities structuration. Using an LDA score (Supplementary Data, Figure S5), the taxa contributing to the monthly evolution of biofilm bacterial communities were highlighted. It appears that a majority of taxa are affiliated with the second month of the experiment, which is coherent with what was obtained with the ß-diversity analyses (Figure 2) but also with a significant increase in the *Cellvibrionaceae* family relative abundance (+46.5%) in the biofilm samples from the control tank in the second month of the experiment (Wilcoxon test, *p*-value = 0.0005). Moreover, this augmentation had a great impact on the bacterial communities present in the biofilm, with an LDA effect size score of 4.15. Some taxa were also associated with other months of the experiment, with bacteria from the order *Micavibrionales* for the first month (Wilcoxon test, *p*-value = 0.003; LDA effect size: 3.93) and from the genus *Blastospirellula* for the fourth month (Wilcoxon test, *p*-value = 0.0007; LDA effect size: 3.95).

### 3.3. Effects on Flat Oyster Mortality and Modulation of Environmental Bacterial Communities Induced by Pseudoalteromonas Supplementation

#### 3.3.1. Innocuity of *Pseudoalteromonas* Strains

At the end of the experiment, the observed survival scores of the flat oysters were 86% for the control tank and for the h*Oe*-125-exposed tank, 82% for flat oysters from the RA15^T^-exposed tank and the h*Cg*-42-exposed tank, and finally, 81% for the flat oysters in the 3J6-exposed tank (Supplementary Data, Figure S6). Statistical tests showed that flat oysters did not present a significantly higher mortality in tanks where *Pseudoalteromonas* strains were added (chi-squared test: *p*-value = 0.07).

#### 3.3.2. Seawater Bacterial Community Preservation

Regarding the bacterial communities found in seawater, the addition of *Pseudoalteromonas* strains to the rearing tanks had no impact on these communities in terms of richness, diversity (α-diversity analyses; ANOVA test: *p*-value > 0.05), or structure (β-diversity analyses; PERMANOVA test: *p*-value > 0.05). However, several effects were observed on biofilm bacterial communities.

#### 3.3.3. Effects on Taxonomical Composition of Biofilms

**Figure 4:**
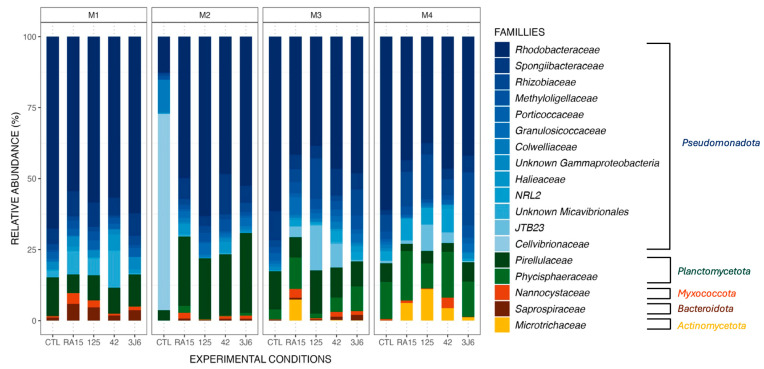
Relative abundance (%) of the major ASVs found in biofilm samples from all experimental conditions (CTL, RA15 = RA15^T^, 125 = h*Oe*-125, h*Cg*-42, 3J6) at each sampling month (M1 to M4). ASVs are represented at the family taxonomic rank. Together, the ASVs shown here comprise 99.6% of all observed ASVs.

Biofilms formed in the tanks supplemented by different *Pseudoalteromonas* strains presented the same main phyla as the control tank (Figure 3) during the four-month experiment, including *Pseudomonadota*, *Planctomycetota*, and *Bacteroidota* (Figure 4). However, the *Microtrichaceae* family from the phylum of *Actinomycetota* was found only in the tanks where the strains were added. Biofilms collected from the RA15^T^-exposed tank in the third month contained this family at a relative abundance of 7.5%, while it was absent in other experimental conditions. By the fourth month, the *Microtrichaceae* family was present in all tanks exposed to *Pseudoalteromonas* strains, with varying relative abundances of 6.2% (RA15^T^), 11.1% (h*Oe*-125), 4.4% (h*Cg*-42), and 1.2% (3J6). Additionally, variations in relative abundance were observed across the experimental conditions, with the same bacterial families present but exhibiting different relative abundances in each condition. For instance, the *Rhodobacteraceae* family, dominant in all biofilm samples, exhibited a relative abundance of 67.7% in the control tank during the first month of the experiment. In comparison, it was present at 54.4% in the RA15^T^-exposed tank, 58.4% in h*Oe*-125, 56.8% in h*Cg*-42, and 62.2% in the 3J6-exposed tank.

These relative abundance differences between each experimental condition did not result in differences in the richness and diversity of the bacterial communities present. The supplementation of the tanks with *Pseudoalteromonas* strains did not affect the richness of the communities present when considering all months (α-diversity analyses, ANOVA test on observed richness, *p*-value > 0.05). The average diversity of bacterial communities throughout the experiment was higher in biofilms from the 3J6-exposed tank compared to the control (post hoc Tukey test on the Shannon index, *p*-value = 0.008) but otherwise non-significant for the tanks exposed to the other *Pseudoalteromonas* strains (post hoc Tukey test on the Shannon index, *p*-value > 0.05).

#### 3.3.4. *Pseudoalteromonas* Strain Supplementation Induces a Shift in Bacterial Communities’ Structure

**Figure 5:**
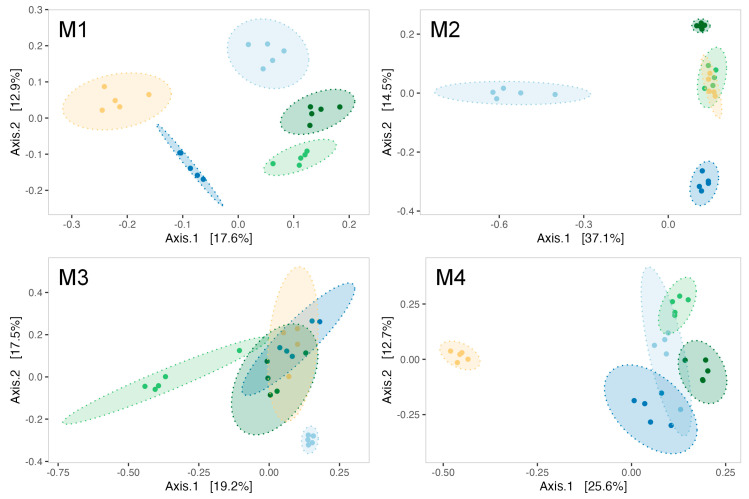
Principal coordinate analysis (PCoA) using Bray–Curtis distance for biofilms formed at every month of the experiment (**M1**–**M4**). Ellipses are drawn for each experimental condition (light blue = CTL, dark blue = RA15^T^, light green = h*Oe*-125, dark green = h*Cg*-42, yellow = 3J6) at a 95% confidence level for a multivariate t-distribution.

ß-diversity analyses indicated a modulation of biofilms’ microbial communities by *Pseudoalteromonas* strain treatments as early as the first month of the experiment (PERMANOVA test, R^2^ = 0.455, F = 4.17, *p*-value = 0.001). Indeed, five distinct ellipses represented each treatment during analysis in the first month (Figure 5M1). The effect of *Pseudoalteromonas* supplementation on the bacterial communities of biofilm was evidenced throughout the maturation with a significant PERMANOVA for each month (Supplementary Data, Table S2). Notably, the bacterial communities in the control tanks differ from those in the strain-exposed tanks during the second (Figure 5M2) and third months (Figure 5M3) of the experiment. By the fourth month, the 3J6-exposed tank distinctly separated from all other experimental conditions (Figure 5M4).

#### 3.3.5. Biofilm Biovolumes Exposed to Pseudoalteromonas Strains

**Figure 6:**
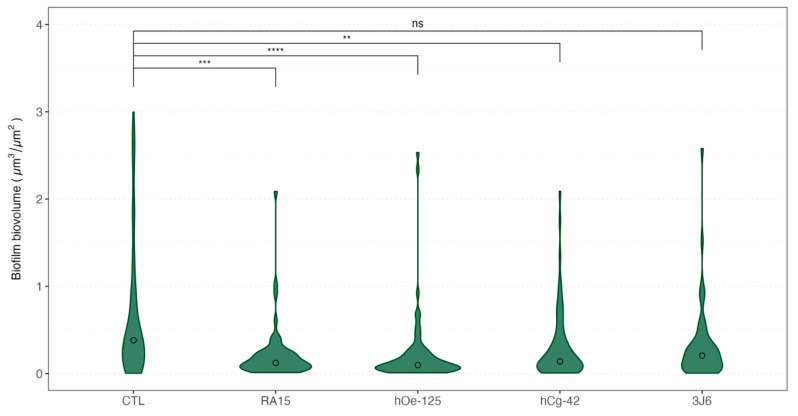
Violin boxplot of the biovolume of the biofilms formed depending on the treatment for all sampling times. Medians values were indicated with a circle for each boxplot. A non-parametric Kruskal–Wallis test was conducted on biovolume median values, followed by post hoc Dunn testing, the results of which are depicted in this figure. Significative codes: *p*-value = [0, 0.0001] (****), *p*-value = [0.0001, 0.001] (***), *p*-value = [0.001, 0.01] (**), ns: non significant.

The biovolume of the biofilm formed during the maturation was also recorded each month. Weekly bathing with all *Pseudoalteromonas* strains, except for the 3J6 strain, led to a significant reduction in biofilm biovolume (Figure 6) during oyster maturation.

**Figure 7:**
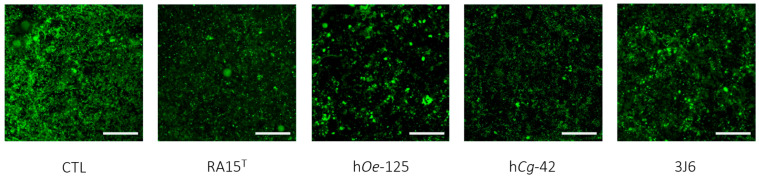
Top-down views of biofilms stained with SYTO-9 and examined using confocal laser scanning microscopy (63× magnification). Scale bars represent 10 µm.

Indeed, as an average for the whole experimentation, a mean biovolume value of 0.776 ± 1.145 µm^3^/µm^2^ was obtained for the control condition. The mean biofilm biovolume value was significantly reduced for three *Pseudoalteromonas* strain treatments of 0.221 ± 0.331 µm^3^/µm^2^ (RA15^T^), 0.230 ± 0.429 µm^3^/µm^2^ (h*Oe*-125), and 0.310 ± 0.409 µm^3^/µm^2^ (h*Cg*-42). However, the 3J6 strain treatment showed a non-significant biovolume reduction with a mean value of 0.328 ± 0.459 µm^3^/µm^2^. These analyses were determined from biofilm images taken by CLSM (Figure 7).

## 4. Discussion

This study provides the first insights into environmental bacterial communities in the context of flat oyster farming during sexual maturation. Indeed, we investigated the effects of weekly bathings with different strains of *Pseudoalteromonas* on bacterial communities in surrounding seawater and biofilms during a complete sexual maturation cycle (4 months) of the flat oyster *Ostrea edulis*. The bacterial communities were analyzed monthly using 16S rRNA gene metabarcoding.

### 4.1. Biofilm vs. Seawater: Unveiling the Differences in Bacterial Communities

Marine biofilms formed during the maturation of the flat oyster were primarily composed of bacterial communities belonging to the phyla *Pseudomonadota* and, to a lesser extent, *Planctomycetota*. The phylum *Pseudomonadota* stands as the predominant phylum in marine biofilms [53,54,55]. The phylum *Planctomycetota* was also commonly identified in bacterial communities of biofilms but in lower relative abundances [56,57]. In surrounding seawater, bacterial communities were also dominated by the phylum *Pseudomonadota* and more specifically by bacterial communities from the classes *Alphaproteobacteria* and *Gammaproteobacteria*. The phylum *Pseudomonadota* precedes *Bacteroidota* in seawater bacterial communities, which were also the main phyla observed in several studies [58,59].

α- and ß-diversity analyses revealed obvious dissimilarities between biofilm and seawater bacterial communities. Thus, in biofilms, bacterial communities showed greater richness and diversity than the ones observed in seawater (α-diversity). Biofilm bacterial communities also presented unique community structuring dynamics (ß-diversity). Such differences have already been reported in various aquatic environments, such as seawater from natural environments, freshwater, and aquaculture hatcheries [60,61,62].

Various parameters have been hypothesized to explain such differences in bacterial community structuration between biofilm and seawater, including environmental factors [54,60,63]. All of these dissimilarities may indeed result from selective pressure and the ability of bacterial communities to form biofilms [64].

### 4.2. Structural Modifications of Biofilm and Seawater Bacterial Communities over Time

Temporal dynamics were observed in the structuring of bacterial communities in surrounding seawater and biofilms sampled from flat oyster rearing. Such dynamics have also been described in tank seawater of the blue shrimp larvae, *Penaeus stylirostris*, where significant daily variations were reported [58]. Nevertheless, in our study, the time scale (i.e., 4 months) covers a longer period than the one used in the available literature.

A structuration of biofilm bacterial communities over time has also been observed during the maturation of *Ostrea edulis*, as well in open sea environments [61] and algaculture tanks [60]. Biofilms formed during the maturation of *Ostrea edulis* showed an increased richness index overtime, corresponding to an increased number of distinct ASVs. However, no significant changes were observed regarding diversity (α-diversity analyses). This observation was consistent with other biofilm bacterial community studies observing the same phenomenon regarding not only richness but also diversity [61,62]. Moreover, it appears that environmental biofilms harbored a succession of bacterial communities associated with different stages of biofilm biovolume in marine environments; these variations have been observed over short as well as prolonged periods [61,62,65].

### 4.3. Modulation of Biofilm by Pseudoalteromonas Strains

We also explored the impact of different *Pseudoalteromonas* strains on the composition of bacterial communities in biofilm and seawater. The weekly bathings with *Pseudoalteromonas* strains impacted the bacterial communities’ structures (Figure 5) without modifications of the richness nor diversity. The weekly introduction of *Pseudoalteromonas* strains into the tanks indeed only influenced the relative abundance of the ASVs present. On the other hand, using CLSM, we observed a significant effect on biofilm. Indeed, the weekly addition of the *Pseudoalteromonas* strains resulted in a significant reduction in biofilm biovolume formed during a 4-month-long sexual maturation of *Ostrea edulis* (Figure 6). The observed reduction in biofilm development by *Pseudoalteromonas* strains was unexpected for two primary reasons. First, an antagonistic effect against specific bacterial components within the biofilm was a plausible hypothesis. However, this is contradicted by the consistent richness and diversity observed over time within the tanks. Second, *Pseudoalteromonas* colonization of the biofilm was anticipated, yet no evidence of this was observed throughout the experiment, as the relative abundance of the *Pseudoalteromonadaceae* family remained unchanged. Therefore, the mechanism(s) by which these strains suppress biofilm formation without compromising its richness and diversity warrant(s) further investigation. As the sequencing method does not allow us to discriminate between the species in presence, many hypotheses can be put forward to explain the phenomenon observed. Amongst those hypotheses, one can think of competition with biofilm-forming bacteria for nutrient availability [66] or quorum-quenching ability, which can prevent the formation of the biofilm [67]. Nonetheless, these strains constitute a biocontrol tool for biofilm and represent a natural treatment that could be advantageous to the aquaculture sector, as biofilm presence remains an ecological and economic issue [55]. Furthermore, the molecular mechanism(s) involved in biofilm volume reduction remains to be clarified.

## 5. Conclusions

The present study demonstrated that the biovolume of biofilm was significantly reduced in the presence of three of the four *Pseudoalteromonas* strains assayed compared to the non-exposed tank, while the diversity and richness of the biofilm microbial communities remained unaffected. The mechanisms behind this observation may involve these specific strains used but also the host’s microbiota, the host itself, and therefore the holobiont. Regardless, *Pseudoalteromonas* strains seem to act as biological agents that modify environmental bacterial communities without causing significant mortality. Given the tangible benefits, including reduced cleaning time and costs, and for the environment through decreased biocide use, these strains could find applications in aquacultures as production additives or even probiotics.

## Data Availability

16S amplicon sequences used for metabarcoding analysis have been made available on the NCBI Sequence Read Archive (SRA) under the BioProject accession reference PRJNA1135127.

## Supplementary Materials

The following supporting information can be downloaded at: https://www.mdpi.com/article/10.3390/microorganisms13020363/s1, Figure S1: Rarefaction curve of all biofilm and seawater samples (n = 120) used in this study; Figure S2: α-diversity measures comparing biofilm and seawater samples of the control condition; Figure S3: Non-metric multidimensional scaling (NMDS) using Bray–Curtis distance for bacterial communities from biofilm or seawater samples from the control condition; Figure S4: Observed richness and diversity (Shannon and InvSimpson indexes) in biofilm samples for each month and in the control tank; Figure S5: LDA displaying biomarkers of each sampling month of biofilms; Figure S6: Kaplan–Meier survival curve of flat oysters from the tank treated with Pseudoalteromonas strains and the control tank; Table S1: Analysis of variance (ANOVA) test comparing sampling groups from the control condition for different α-diversity measures. Table S2: Permutational analysis of variance (PERMANOVA) to test the impact of strains on biofilms formed for each month of the experiment using the Bray–Curtis distance.
