# Supplementary figures and images for: Pseudoalteromonas Strains as Biofilm Control Agents in Ostrea edulis Aquaculture: Reducing Biofilm Biovolume While Preserving Microbial Diversity
Source: Microorganisms. 2025 Feb 7;13(2):363. doi: 10.3390/microorganisms13020363 (PMC11858371; doi:10.3390/microorganisms13020363)

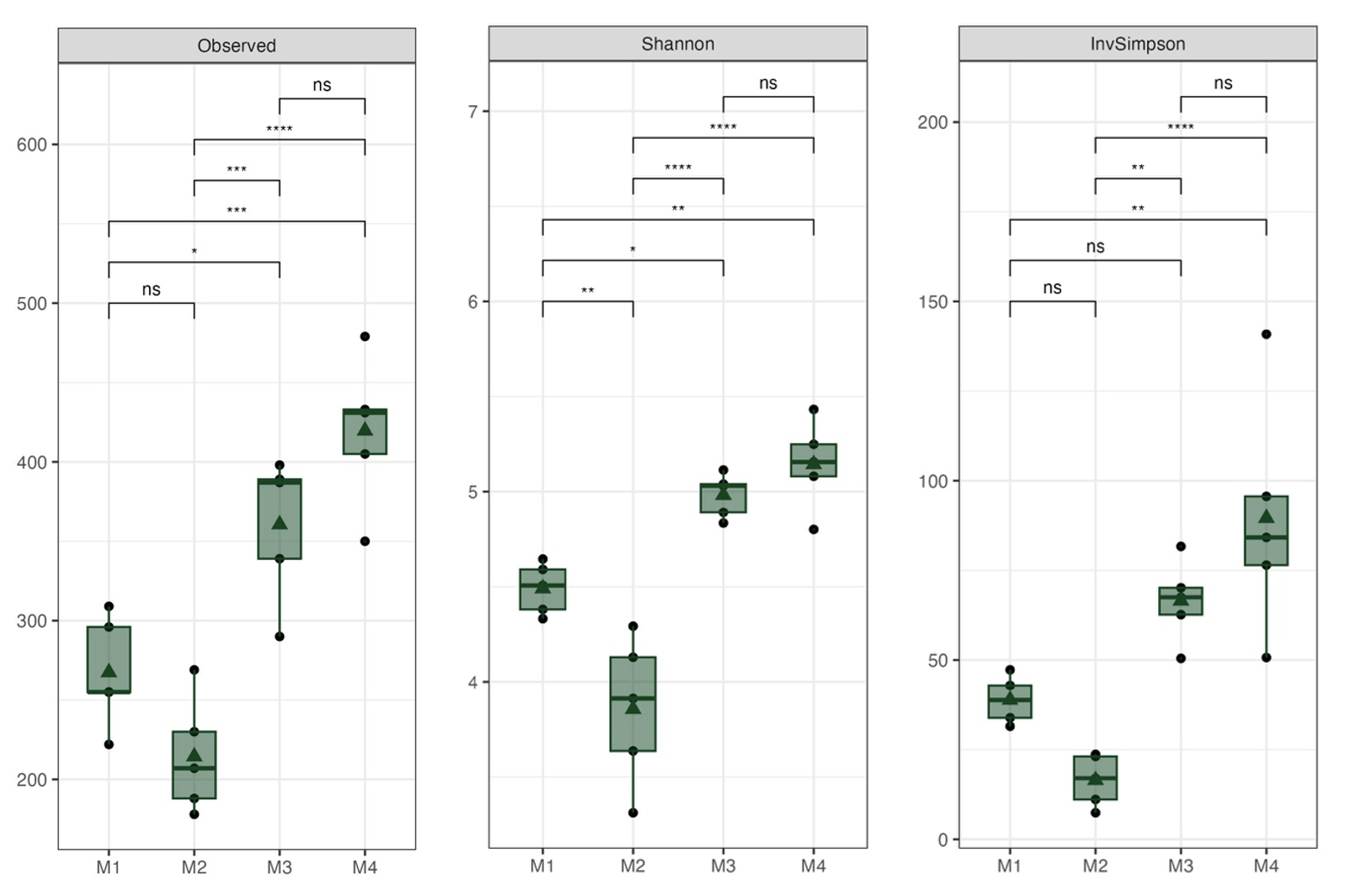

Supplement: Supplementary file 1 [file microorganisms-13-00363-s001.zip › SUPPLEMENTARY-DATA/Figure-S4.png]

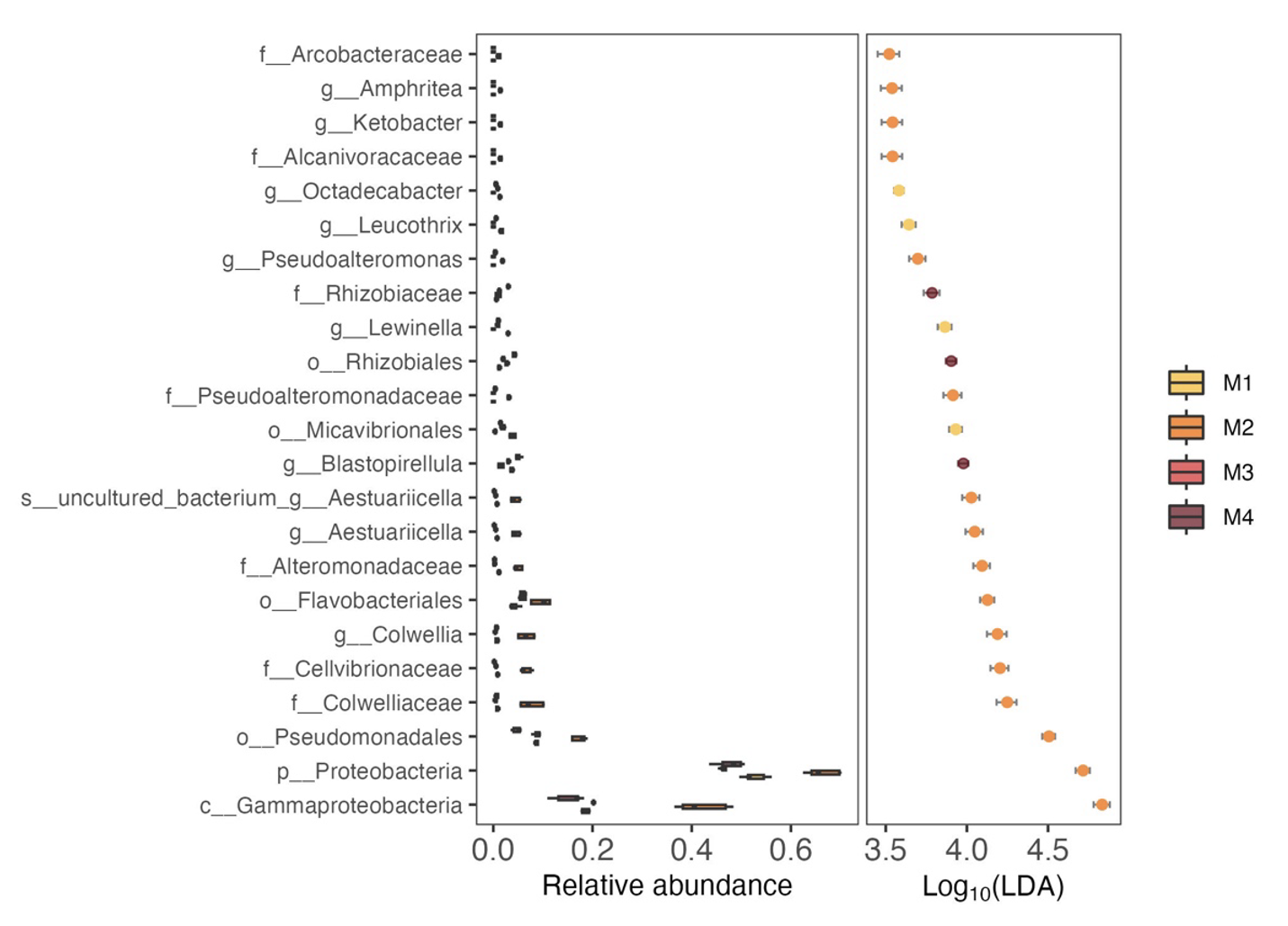

Supplement: Supplementary file 1 [file microorganisms-13-00363-s001.zip › SUPPLEMENTARY-DATA/Figure-S5.png]

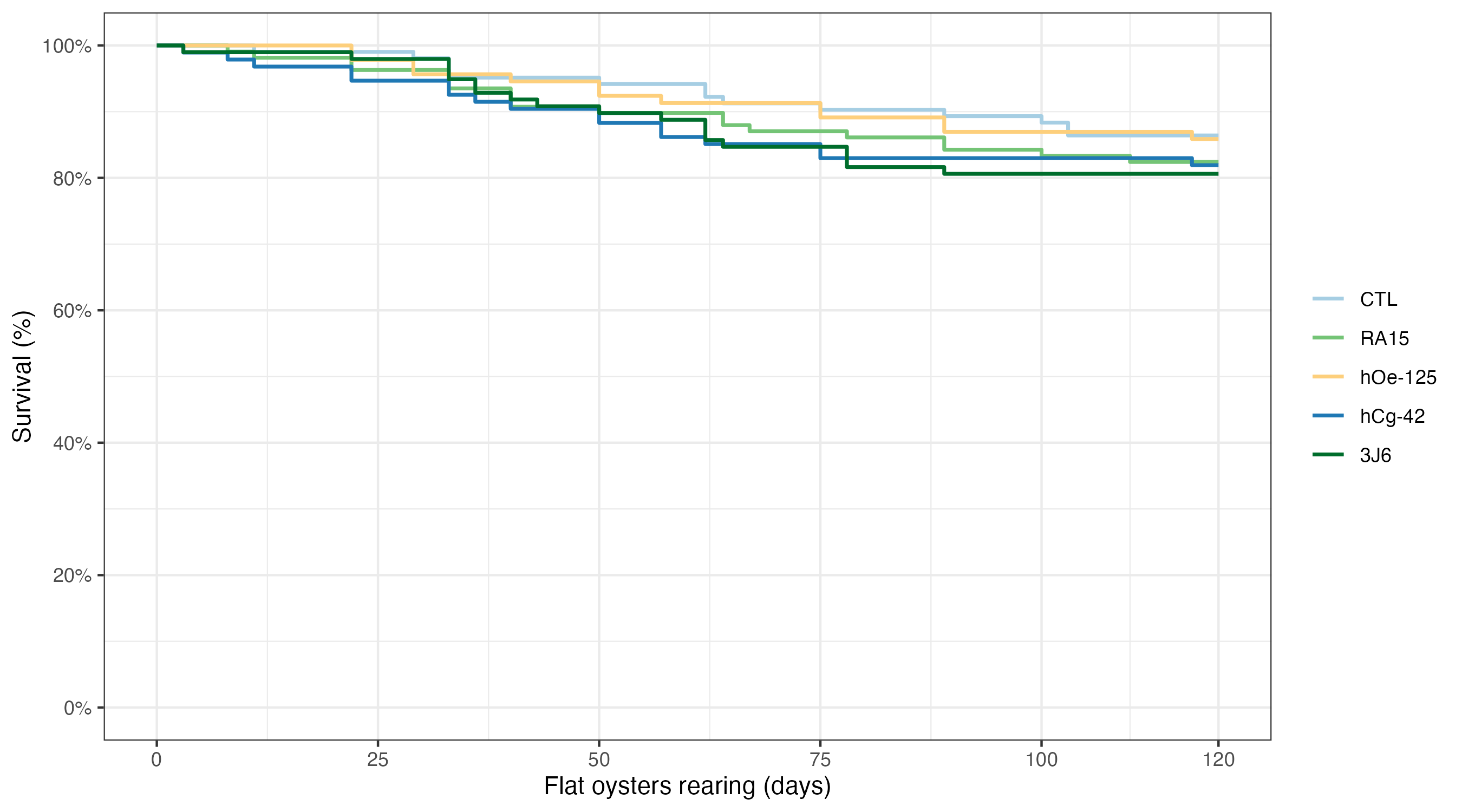

Supplement: Supplementary file 1 [file microorganisms-13-00363-s001.zip › SUPPLEMENTARY-DATA/Figure-S6.png]

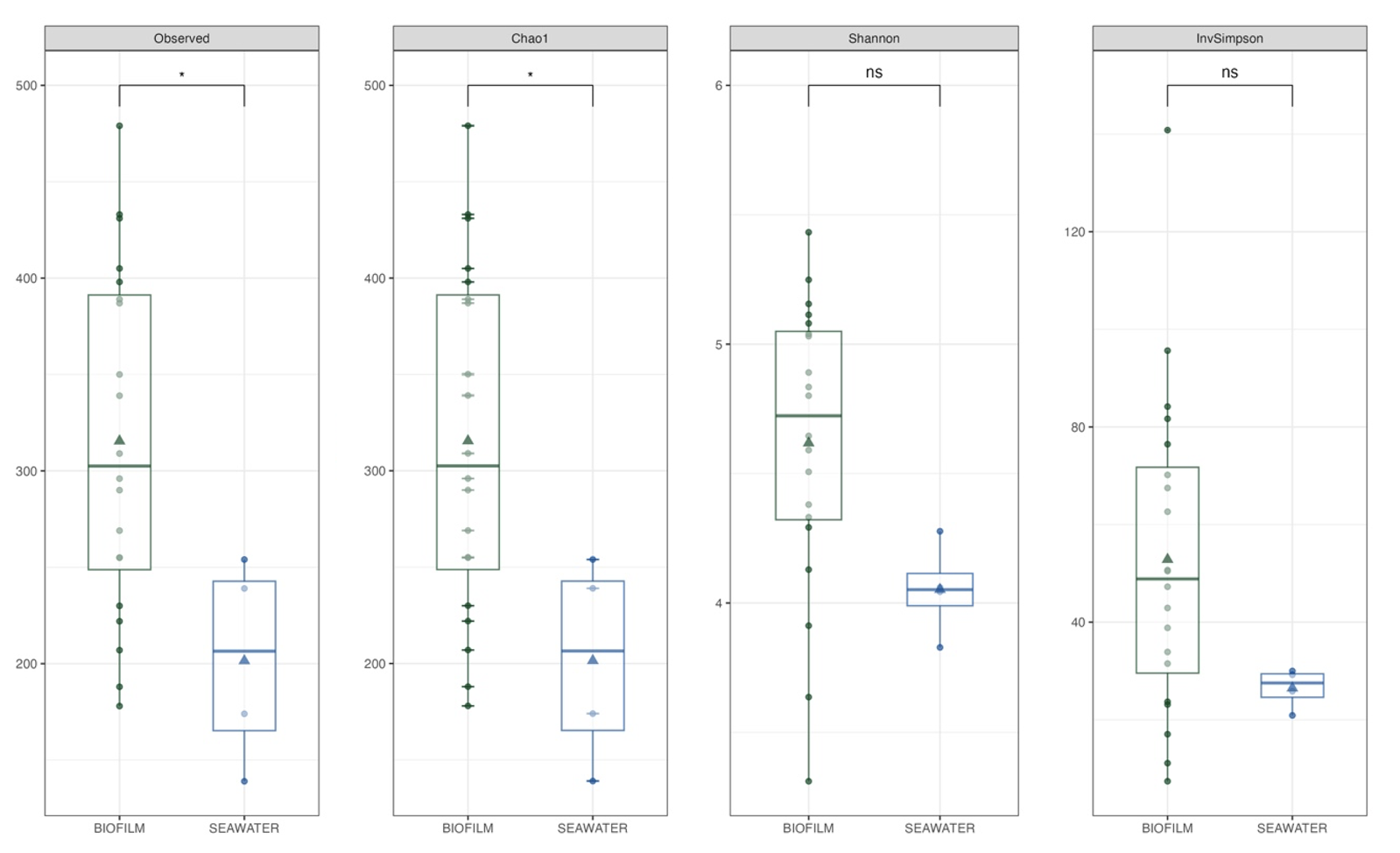

Supplement: Supplementary file 1 [file microorganisms-13-00363-s001.zip › SUPPLEMENTARY-DATA/Figure-S2.png]

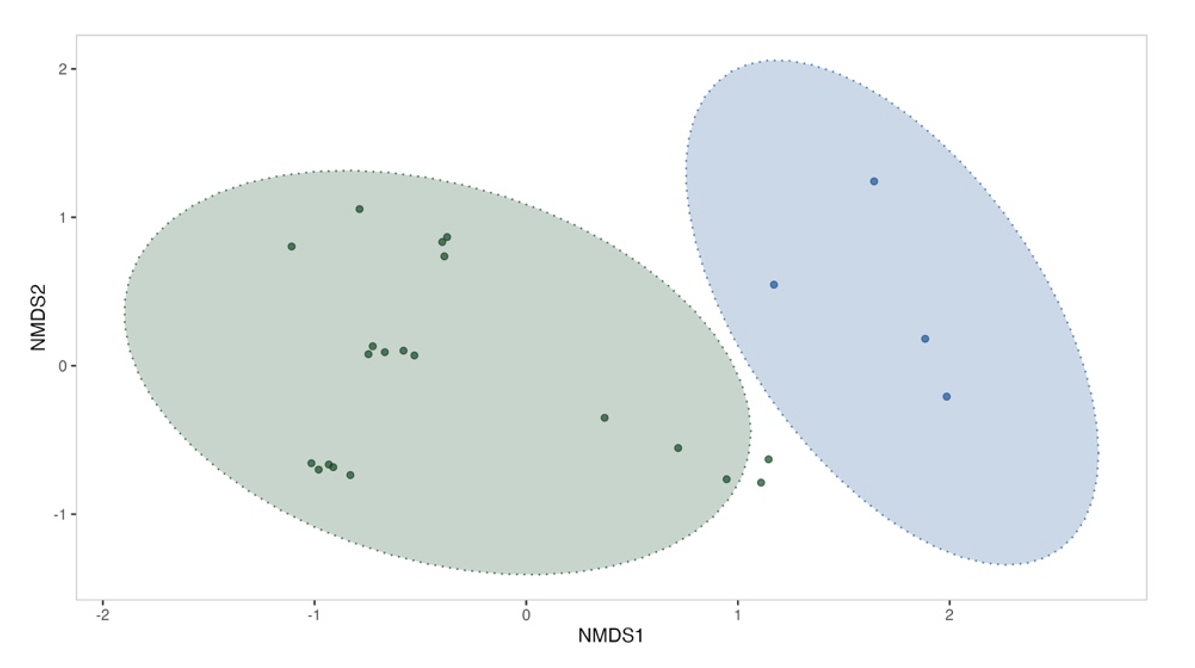

Supplement: Supplementary file 1 [file microorganisms-13-00363-s001.zip › SUPPLEMENTARY-DATA/Figure-S3.png]

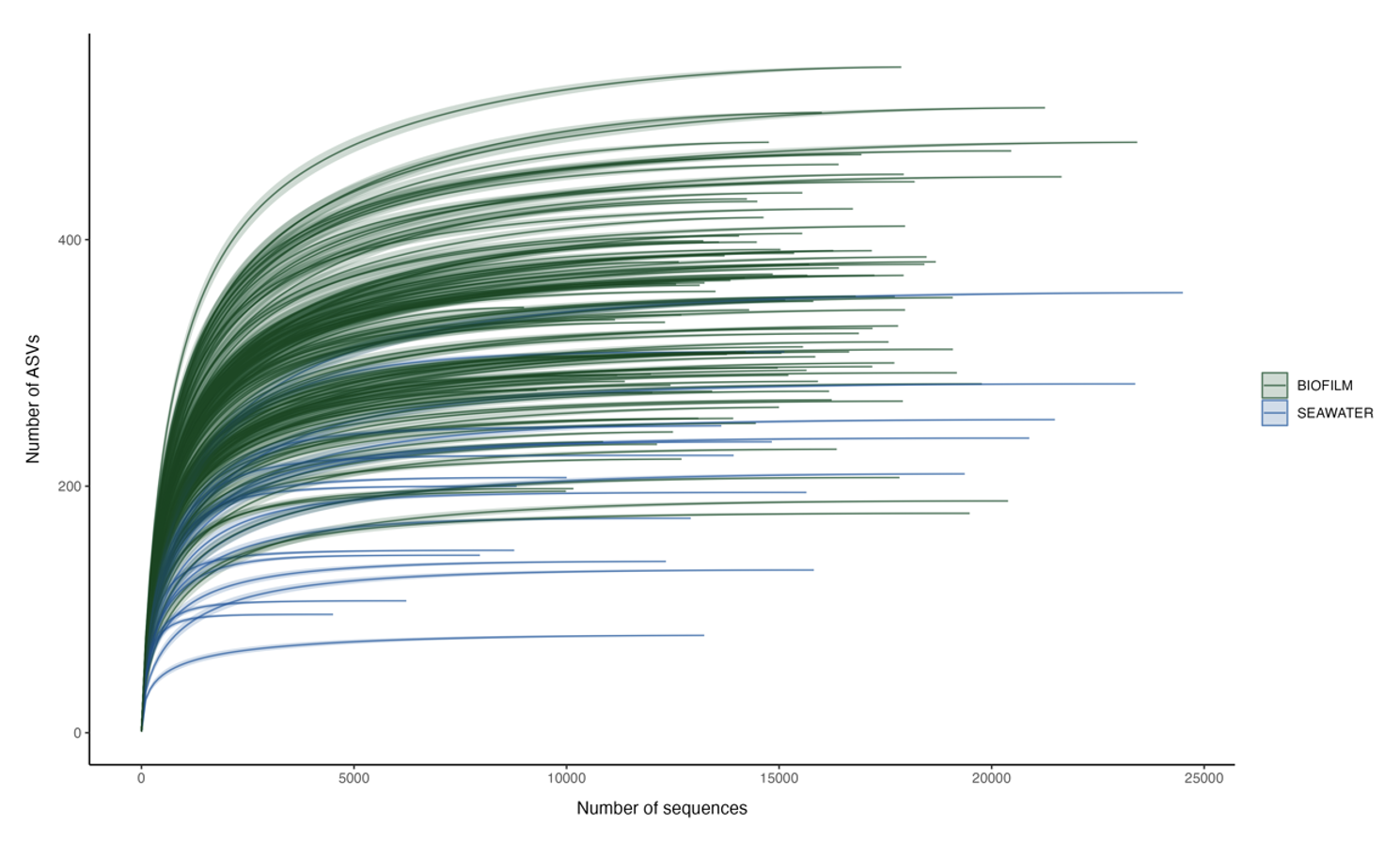

Supplement: Supplementary file 1 [file microorganisms-13-00363-s001.zip › SUPPLEMENTARY-DATA/Figure-S1.png]

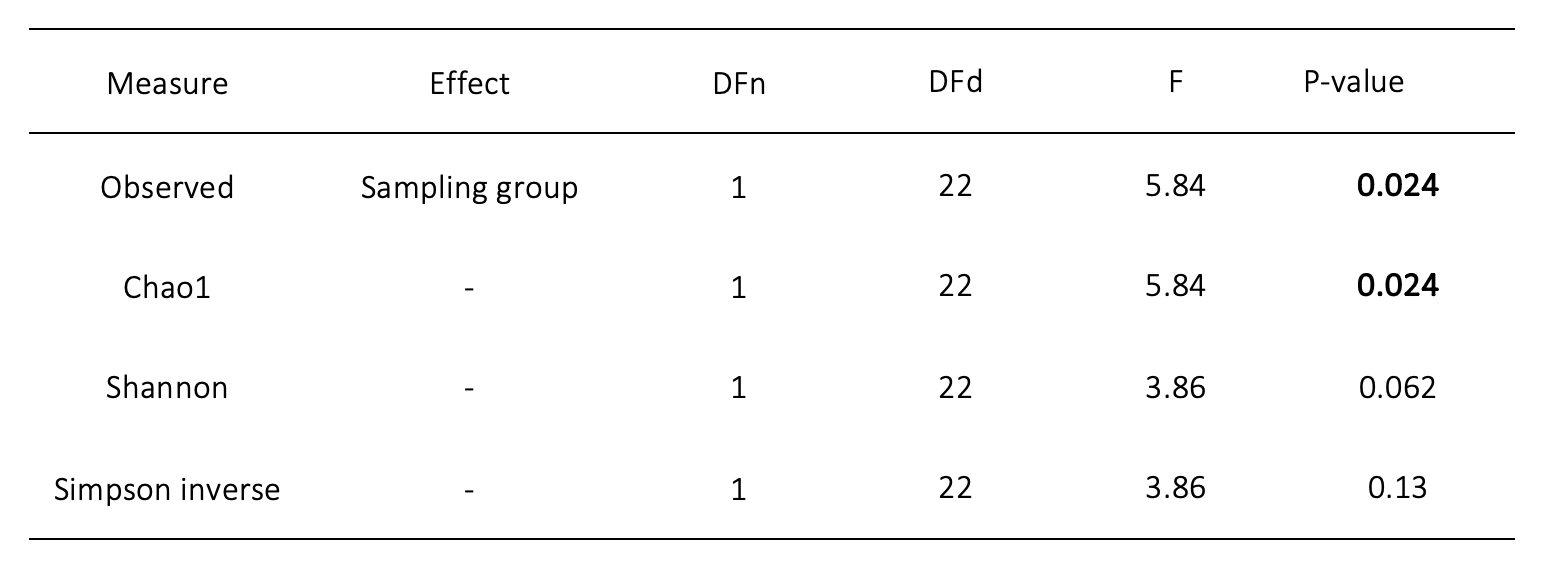

Supplement: Supplementary file 1 [file microorganisms-13-00363-s001.zip › SUPPLEMENTARY-DATA/Table-S1.png]

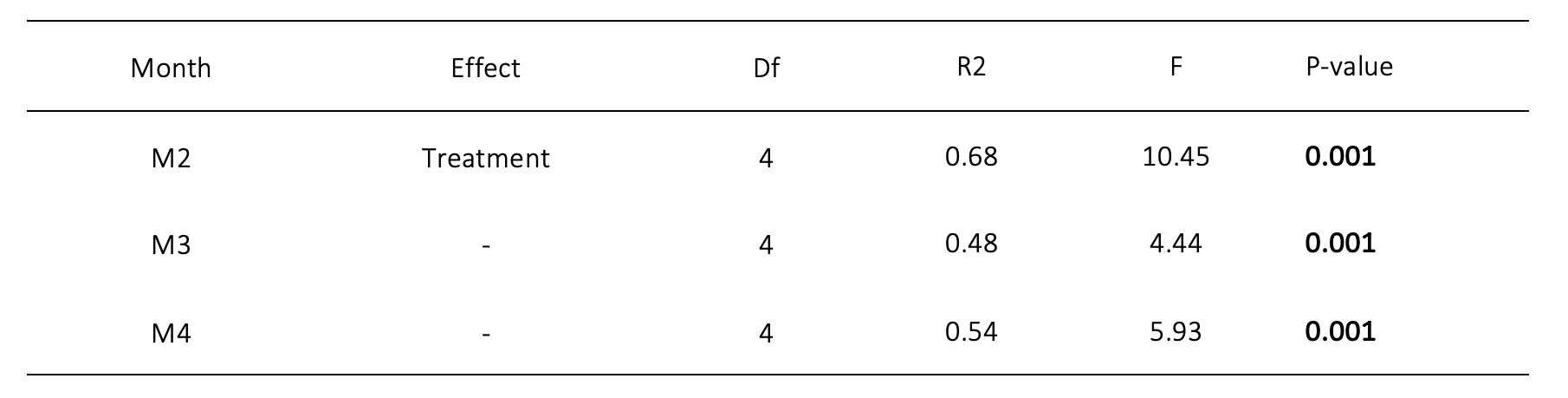

Supplement: Supplementary file 1 [file microorganisms-13-00363-s001.zip › SUPPLEMENTARY-DATA/Table-S2.png]
